# Abnormal Production of Pro- and Anti-Inflammatory Cytokines by Lupus Monocytes in Response to Apoptotic Cells

**DOI:** 10.1371/journal.pone.0017495

**Published:** 2011-03-14

**Authors:** Sangeeta Sule, Antony Rosen, Michelle Petri, Ehtisham Akhter, Felipe Andrade

**Affiliations:** 1 Department of Pediatrics, Johns Hopkins University School of Medicine, Baltimore, Maryland, United States of America; 2 Department of Medicine, Johns Hopkins University School of Medicine, Baltimore, Maryland, United States of America; 3 Department of Pathology, Johns Hopkins University School of Medicine, Baltimore, Maryland, United States of America; Institut Jacques Monod, France

## Abstract

Monocytes are a key component of the innate immune system involved in the regulation of the adaptive immune response. Previous studies have focused on apoptotic cell clearance abnormalities in systemic lupus erythematosus (SLE) monocytes. However, whether SLE monocytes might express unique patterns of cytokine secretion in response to apoptotic cells is still unknown. Here, we used monocytes from healthy controls and SLE patients to evaluate the production of TNF-α and TGF-β in response to apoptotic cells. Upon recognition of apoptotic material, monocytes from healthy controls showed prominent TGF-β secretion (mean ± SD: 824.6±144.3 pg/ml) and minimal TNF-α production (mean ± SD: 32.6±2.1 pg/ml). In contrast, monocytes from SLE patients had prominent TNF-α production (mean ± SD: 302.2±337.5 pg/ml) and diminished TGF-β secretion (mean ± SD: 685.9±615.9 pg/ml), a difference that was statistically significant compared to normal monocytes (p≤10^−6^ for TNF-α secretion, and p = 0.0031 for TGF-β, respectively). Interestingly, the unique cytokine response by SLE monocytes was independent of their phagocytic clearance efficiency, opsonizing autoantibodies and disease activity. We further showed that nucleic acids from apoptotic cells play important role in the induction of TNF-α by lupus monocytes. Together, these observations suggest that, in addition to potential clearance defects, monocytes from SLE patients have an abnormal balance in the secretion of anti- and pro-inflammatory cytokines in response to apoptotic cells. Since the abnormal cytokine response to apoptotic material in SLE is not related to disease activity and opsonizing autoantibodies, it is possible that this response might be an intrinsic property of lupus monocytes. The studies focus attention on toll-like receptors (TLRs) and their downstream pathways as mediators of this response.

## Introduction

Systemic lupus erythematosus (SLE) is a multisystem autoimmune disease characterized by an exuberant autoantibody response to a wide variety of autoantigens. Disease is the result of a cascade of events (induced by hormonal and environmental factors) that occur on the background of an appropriate genetic predisposition. In SLE, T and B-cell autoimmune responses result in the generation of autoantibodies and immune complexes, along with autoreactive T cells, which together cause pathology in several target organs, including skin, blood vessels, lung and kidney [1]. Defining the mechanisms underlying SLE initiation, flares, and damage remains a high priority. The observation that lupus autoantigens are selectively clustered in apoptotic surface blebs initially focused attention on apoptosis as an important pathway, upstream of inflammatory processes, which may be relevant in initiating and propagating SLE [2], [3].

In tissues, apoptotic cells are rapidly engulfed by macrophages in the early phase of apoptotic cell death, and this uptake is associated with secretion of anti-inflammatory cytokines, such as TGF-β and IL-10, decreased secretion of IL-12 and TNF-α and failure to upregulate co-stimulatory molecules [4]–[8]. Thus, the early and efficient recognition and engulfment of apoptotic cells has been proposed to be necessary to induce tolerance to autoantigens. Although cumulative evidence from experimental animal models has demonstrated that adequate apoptotic cell clearance plays a role in mediating tolerance to apoptotic cells and preventing autoimmunity [9]–[16], in human SLE, the possible pathogenic consequences of an abnormal interaction between apoptotic material and phagocytic cells remains unclear. Persuasive but indirect evidence suggests that patients with SLE have impaired phagocytosis of apoptotic cells [17]–[20], but whether the phagocytic defect is intrinsic to their macrophages, or acquired as result of the abnormal inflammatory lupus environment (i.e. cytokines and/or opsonizing molecules) is still unclear [21], [22]. Paradoxically, lupus autoantibodies facilitate apoptotic cell clearance and interestingly, trigger TNF-α release by macrophages isolated from healthy donors [23]. However, whether SLE phagocytes might have unique patterns of cytokine secretion in response to apoptotic cells has still to be defined.

In this study, we demonstrate that monocytes from SLE patients have an abnormal balance in the secretion of anti- and pro-inflammatory cytokines in response to apoptotic cells. Interestingly, this cytokine response is independent of the phagocytic clearance efficiency, the presence of opsonizing autoantibodies, and lupus disease activity, and it is associated to the presence of nucleic acids in the apoptotic cell milieu. These findings provide novel insights into the pathogenic interface between lupus monocytes and apoptotic cells, and might offer a mechanistic explanation into the unexpected benefit that blocking TNF-α activity has had in the treatment of patients with SLE. In addition, since the abnormal cytokine response to apoptotic material in SLE is not related to disease activity, it is possible that these findings may suggest a primary defect (either genetic or epigenetic) in the immunomodulatory response to apoptotic cells by lupus monocytes.

### Results

### Lupus monocytes produce a pro-inflammatory pattern of cytokine secretion in response to apoptotic cells

**Figure 1:**
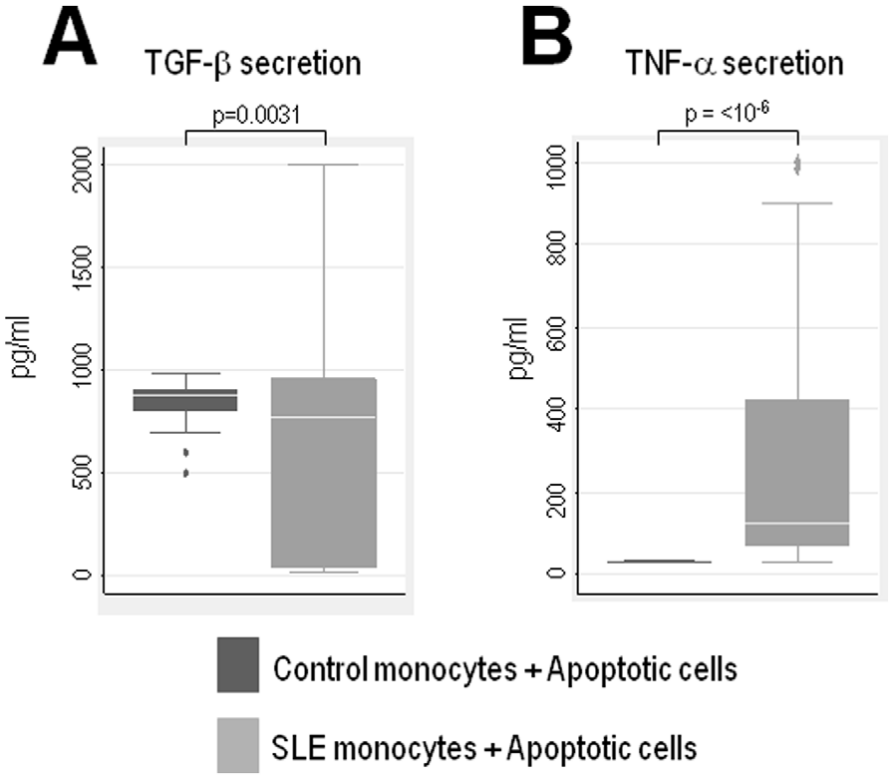
Monocytes from patients with SLE have prominent production of TNF-α, but decreased production of TGF-β. Monocytes obtained from healthy donors (n = 13) or patients with SLE (n = 47) were co-incubated in the presence of apoptotic Jurkat cells. After overnight incubation, production of TGF-β (**A**) and TNF-α (**B**) were determined in the supernatants by ELISA. Data is shown as boxplots. Comparison of mean cytokine secretion between groups was performed by Students t-test.

**Figure 2:**
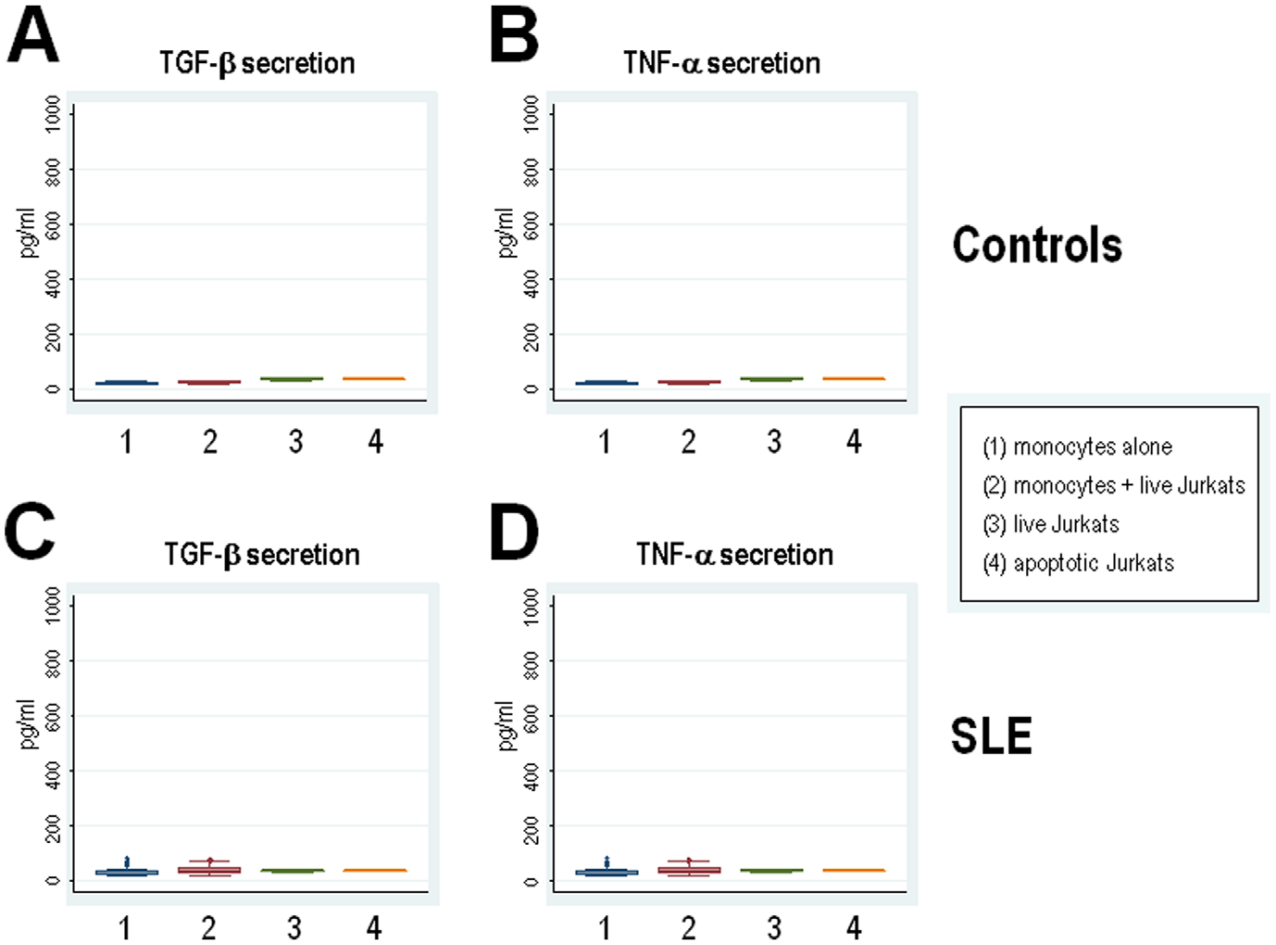
Production of TGF-β and TNF-α by live or apoptotic Jurkat cells, non-stimulated monocytes and monocytes plus live Jurkat cells. Monocytes from healthy donors (A & B) or patients with SLE (C & D) were incubated alone or in the presence of live Jurkat cells. In addition, live or apoptotic Jurkat cells were incubated alone. After overnight incubation, production of TGF-β (A & C) and TNF-α (B & D) were determined in the supernatants by ELISA.

In tissues and *in vitro*, apoptotic cells are rapidly engulfed by macrophages and this uptake is associated with enhanced secretion of TGF-β and minimal production of TNF-α. To determine whether SLE patients have unique patterns of cytokine secretion in response to apoptotic material, we studied a large number of patients (47 patients) and quantified the production of TGF-β and TNF-α using a highly standardized and reproducible assay in which control or SLE monocytes are co-incubated in the absence or presence of dying cells in which apoptosis was induced by ultraviolet B (UVB)-irradiation. Since autoantibodies enhance the recognition and binding of apoptotic material [23], the assays were performed using commercial human AB serum to directly address the effect of apoptotic cells in monocytes in the absence of autoantibodies. Thus, as previously described [4], upon recognition of apoptotic material, normal monocytes are characterized by prominent TGF-β secretion (mean ± SD: 824.6±144.3 pg/ml) and minimal TNF-α production (mean ± SD: 32.6±2.1 pg/ml) (Figure 1A and 1B, respectively). Interestingly, when monocytes from SLE patients were exposed to apoptotic cells, the pattern of cytokine secretion was strikingly different compared to control cells. Thus, SLE monocytes were characterized by prominent TNF-α production (mean ± SD: 302.2±337.5 pg/ml) and diminished TGF-β secretion (mean ± SD: 685.9±615.9 pg/ml) in response to apoptotic cells, a difference that was statistically significant compared to normal monocytes (p≤10^−6^ for TNF-α secretion, and p = 0.0031 for TGF-β, respectively) (Figure 1A and 1B, respectively). The basal production of TGF-β and TNF-α in non-stimulated monocytes from controls (Figure 2A and 2B, respectively) or SLE patients (Figure 2C and 2D, respectively) was not statistically different, and neither apoptotic Jurkat cells alone, monocytes incubated with live Jurkat cells, or live Jurkat cells alone, produced and/or induced TGF-β (Figure 2A and 2C) and/or TNF-α secretion (Figure 2B and 2D). Together, these data demonstrate that the pattern of cytokine production generated by monocytes upon recognition of apoptotic material is quite distinct in patients with SLE compared to healthy monocytes. In addition, it suggests that this response is not dependent on the presence of opsonizing autoantibodies. It is noteworthy that the range of TGF-β and TNF-α secretion by lupus monocytes was quite heterogeneous among the patients (Table 1). Thus, although 57% of the SLE patients showed decreased production of TGF-β, the rest showed normal (28%) or even high (15%) TGF-β secretion compared to monocytes from healthy controls. In the case of TNF-α, almost 90% of the lupus monocyes secreted TNF-α above the normal range; the magnitude of this increase varied widely among the patients. Interestingly, we found no association between the production levels of TGF-β and TNF-α by lupus monocytes and disease activity (determined by SLEDAI), treatment, laboratory or clinical features, or demographic characteristics in the patients (Table 1 and data not shown). Taken together, these data strongly suggest that the abnormal response of lupus monocytes to apoptotic cells may result from an intrinsic property of the SLE monocyte itself.

**Table 1:** Range of TNF-α and TGF-β secretion by lupus monocytes upon activation with apoptotic cells.

TGF-β (pg/ml)	SLE N (%)	SLEDAI score§	P-value
<391.6	27 (57.4)	3.9	–
**391.7–1257.5** *	13 (27.7)	4.1	0.65
>1257.5	7 (14.9)	4.4	0.81

TNF-α (pg/ml)	SLE N (%)	SLEDAI score§	P-value
**26.3–38.9** *	5 (10.6)	4.2	–
39–100	11 (23.4)	4.5	0.78
100–500	20 (42.5)	4.4	0.81
>500	11 (23.4)	4.5	0.83

*Normal range of cytokine secretion based on the mean ± 3SD from healthy controls.

§ Mean SLEDAI scores were compared using t-test for comparison of means, reference group was the lowest secreting patients.

### Monocyte phagocytosis of apoptotic cells and TNF-α production

**Figure 3:**
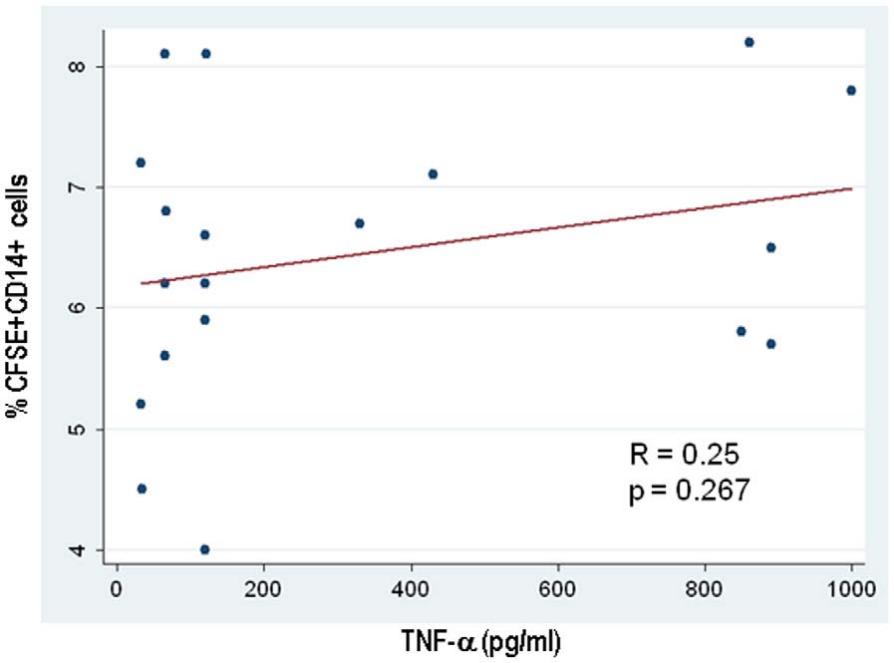
Pearson's correlation between phagocytosis of apoptotic cells and TNF-α production by SLE monocytes. Control (Figure S1) or SLE monocytes were co-incubated with CFSE-labeled apoptotic Jurkat cells. The percentage of cells positive for both CD14 and CFSE was used to quantify phagocytosis of apoptotic Jurkat cells by monocytes. Only data from SLE monocytes is shown. Statistical analysis was performed with Stata 10 software (Stata Corporation, College Station, TX).

Impaired clearance of apoptotic material has been proposed as a mechanism underlying SLE pathogenesis. Although the decrease in the production of TGF-β by SLE monocytes could be explained by a defect in the recognition and engulfment of apoptotic cells, this model does not explain the prominent production of TNF-α observed in the patients. To gain insights into the relation between apoptotic cell clearance and TNF-α secretion, we quantified the apoptotic clearance capacity as well as TNF-α production in controls and SLE patients. Thus, monocytes from 8 healthy donors and 20 SLE patients were co-incubated in the presence of CFSE-labeled live and apoptotic Jurkat cells, and phagocytosis was determined by the percentage of cells positive for both CD14 and CFSE (Figure S1). In the presence of live Jurkat cells, the percentage of phagocytosis by control or SLE monoctyes was minimal (range: 1.1–2.1% and 1.2–2.3%, respectively). In contrast, both control and SLE monocytes were capable of phagocytosing apoptotic Jurkat cells. However, the difference was not statistically significant between the 2 groups (normal controls: 6.3±1.1% vs. SLE patients: 6.4±1.1%, p = 0.70) (Table S1). There was a wide range in TNF-α cytokine secretion in the SLE patients (31–1000 pg/ml). However, we found no correlation between the clearance capacity and TNF-α secretion by lupus monocytes (p = 0.267, R = 0.26) (Figure 3). Taken together, these data suggest that the abnormal secretion of TNF-α by lupus monocytes is a process independent of apoptotic cell engulfment.

### Nucleic acids from apoptotic cells are important components in the induction of TNF-α secretion by SLE monocytes

**Figure 4:**
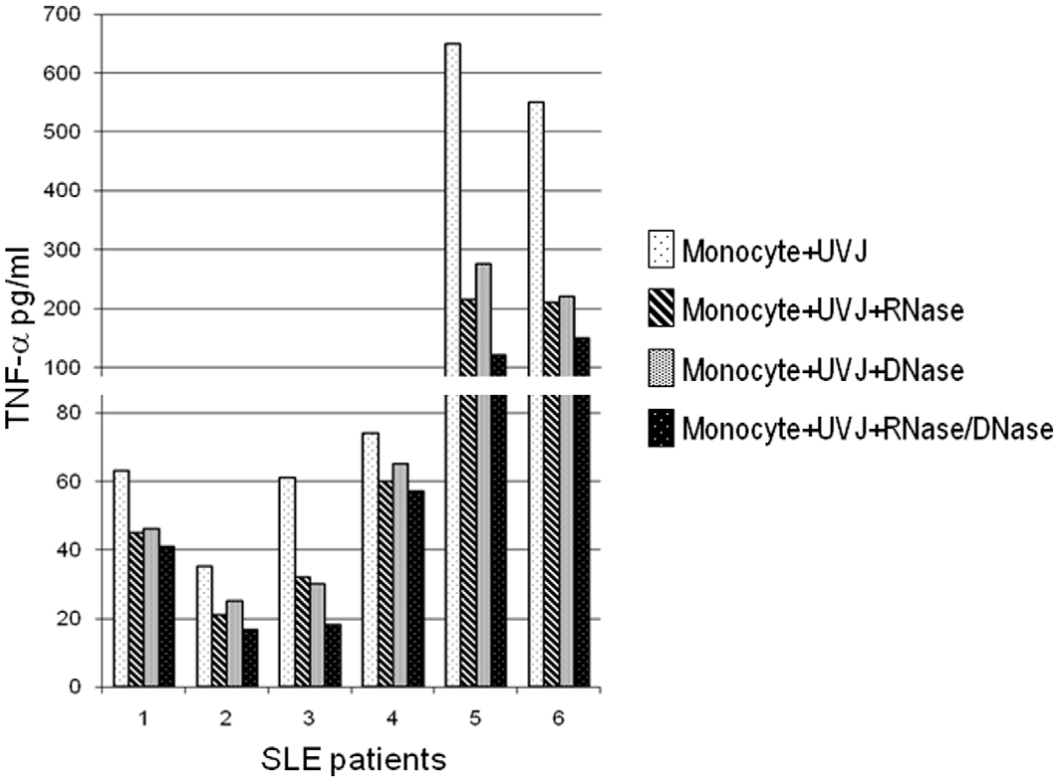
TNF-α production by lupus monocytes in response to apoptotic cells is sensitive to RNase and DNase treatment. Monocytes from six consecutive SLE patients were co-incubated with apoptotic Jurkat cells (UVJ) in the absence or presence of 5 µg/ml of RNase, 16 u/ml of DNase, or both nucleases; and TNF-α production was determined by ELISA in the supernatants.

Beside the signals triggered through the interaction and/or engulfment of apoptotic blebs by monocytes, it is possible that other components from apoptotic cells might be responsible of activating lupus monocytes. In this regard, there is increasing evidence demonstrating that components containing nucleic acids which are exposed to the surface and/or released from UVB-irradiated cells dying by apoptosis are potent activators of the innate immune response [2], [24]–[28]. In order to address whether RNA and/or DNA from the apoptotic cells might be involved in the abnormal induction of TNF-α in SLE monocytes, monocytes from patients with lupus were co-incubated with apoptotic cells, in the absence or presence of RNase A, DNAse I, or both, and TNF-α production in the supernatants was determined. In the presence of apoptotic cells, monocytes from six consecutive SLE patients showed TNF-α production with a variable range above the normal limit observed in controls (i.e.≥39 pg/ml). The presence of RNase A or DNase I decreased the production of TNF-α by SLE monocytes with a more prominent effect in the presence of both nucleases (Figure 4) and interestingly, these results were more striking in those SLE monocytes that were high secretors of TNF-α (Figure 4, patients 5 and 6). In summary, these data demonstrate that monocytes from patients with SLE have an abnormal pro-inflammatory cytokine response to apoptotic material, and strongly suggest that this response is mediated in part through the recognition of nucleic acid containing components from apoptotic cells.

## Discussion

Systemic autoimmune diseases are a complex and heterogeneous group of disorders in which disease may reflect a self-amplifying loop wherein antigen (possibly from apoptotic cells) stimulates the immune system and induces specific inflammatory effector pathways. The subsequent autoimmune phenotype may depend on the source of autoantigen in concert with the specific cytokine profile driven by the antigen. Thus, understanding these effector pathways in unique autoimmune microenvironments might have important implications in term of autoimmune disease pathogenesis as well as the development of rational therapies. In this report, we provide the first evidence that human lupus monocytes have a unique pattern of cytokine secretion in response to apoptotic cells, which is paradoxically characterized by high TNF-α and low TGF-β production.

In SLE, there is cumulative evidence suggesting that apoptotic cells are a source of autoantigens and/or adjuvant signals required to initiate and/or propagate the autoimmune process [3], [28]. Hypothetically, an impaired clearance of apoptotic corpses in lupus might allow apoptotic cells to reach secondary necrosis and subsequent release of intracellular contents. Antigen presenting cells (APCs) may then acquire modified autoantigens and initiate an autoimmune reaction. In this regard, special attention has been focused on understanding the pathways involved in apoptotic cell clearance and how these pathways might be affected in SLE [9]–[20]. However, independently of the clearance capacity of lupus phagocytes, the direct effect that apoptotic cells might have on the function of lupus phagocytes has not been explored. *In vitro*, phagocytes from healthy donors rapidly engulf apoptotic cells and this uptake is associated with secretion of anti-inflammatory cytokines, such as TGF-β and IL-10, decreased secretion of IL-12 and TNF-α[4]–[8]. Interestingly, when normal phagocytes are exposed to apoptotic cells which have been previously opsonized with antiphospholipid antibodies (a common SLE autoantibody), there is a substantial increase in the recognition and uptake of apoptotic cells by macrophages, accompanied by TNF-α secretion [23]. In these studies, it is remarkable that lupus monocytes express a potent inflammatory response to apoptotic material, which is independent of opsonizing autoantibodies, clearance efficiency and lupus disease activity. Although previous studies have demonstrated that lupus monocytes produce substantially higher levels of TNF-α upon stimulation with LPS or immune complexes [29], [30], this is the first study that shows that this abnormal pattern of cytokine production can be induced by apoptotic cells.

The lack of association between disease activity and production of TNF-α by lupus monocytes is not surprising, particularly considering that although numerous reports agree that TNF-α expression is elevated in SLE, the association between TNF-α levels and disease activity is less consistent [29], [31]–[42]. Nevertheless, these findings may suggest that the abnormal cytokine response observed in lupus monocytes may represent an intrinsic property of the monocyte itself. Considering that monocytes from almost 90% of SLE patients showed increased production of TNF-α in response to apoptotic cells, it is likely that several predisposing factors related to TNF-α production might be involved in this process. Thus, for example, known genetic polymorphisms and/or epigenetic changes at the TNF-α locus associated with SLE may contribute to this cytokine pattern in lupus monocytes [30], [43]–[45]. In addition, since nucleic acids are important elements in apoptotic cells that are required for the abnormal cytokine response by lupus monocytes, TLRs (specifically TLR3, 7, 8 and 9) and their downstream pathways may also play important role as mediators of this response. In this regard, different murine models of lupus have been used to examine the role of TLR9/7 in the response against self nucleic acids. Interestingly, it has been found that TLR7 deficiency ameliorated disease whereas TLR9 deletion resulted in more severe disease [46], [47]. Thus, it is possible that unique patterns of TLRs expression may dictate the cytokine response induced by apoptotic cells. In humans, the independent role of TLRs in lupus pathogenesis is less clear [48]–[52]. In a recent study, it was found that the expression of several TLRs (i.e. TLR-2, -3, -4, -5, -6, -8 and -9) were significantly increased in SLE monocytes and interestingly, this pattern of expression was independent of disease activity [52]. Furthermore, genetic variants of *TNFAIP3*, a downstream component recruited by the TLRs and that restricts NF-kB-dependent signaling, has been recently associated with SLE [53], [54]. Thus, independently or synergistically, these and other factors may contribute to the magnitude of TNF-α production by lupus monocytes.

The pathogenic relevance of TNF-α in SLE is not yet clear. While numerous reports agree that TNF-α is overexpressed in patients with SLE [35]–[42] and MRL/lpr mice [55], [56], this is not universal. In some lupus models (e.g. the NZB x NZW F1 mouse), reduced levels of TNF-α are observed, and replacement therapy with recombinant TNF-α induces a significant delay in the development of nephritis [57]. Additionally, there are well-described associations association between TNF-α blockade and the induction of lupus-like disease in humans [58], [59]. One way to define the role for TNF-α pathways in human SLE is to abrogate TNF-α signaling and evaluate effects on disease activity. A recent open-label study of infliximab (a drug which blocks TNF-α activity) in 6 adults with moderately-active SLE reported promising results [60]. In this regard, 3 patients with arthritis experienced remission of joint symptoms and in 4 patients with nephritis, proteinuria decreased within 1 week of infliximab administration and diminished by >60% after 8 weeks of therapy. Interestingly, antibodies to dsDNA and cardiolipin increased in 4 of the 6 patients. While TNF-α is generally considered a potent proinflammatory cytokine, it also suppresses mitogen-induced B cell differentiation and antibody production [61], an effect that might explain both the anti-inflammatory benefit of TNF-α blockade observed in SLE, as well as the increase in autoantibody production.

In summary, we provide evidence that monocytes from patients with SLE have an abnormal production of TNF-α and TGF-β in response to apoptotic cells. It is possible that these findings may result from a primary defect (either genetic or epigenetic) in the immunomodulatory response to apoptotic cells by lupus monocytes.

## Methods

### Ethics

The study was approved by The Johns Hopkins Medicine Institutional Review Board (IRB) and all individuals signed an informed consent.

### Subject Selection

After obtaining IRB approval, 13 healthy controls (6 women and 7 men, mean age 33.7±7.9 years) were recruited and informed consent obtained. Forty-seven SLE patients (43 women and 4 men, mean age 47.4±10.7 years) were recruited through the Johns Hopkins SLE cohort, an ongoing, NIH-funded prospective study. There are well-established protocols and data collection sheets completed at each patient visit, including laboratory tests (complete blood count, sedimentation rate, autoantibodies, complement levels) and clinical data.

### Preparation of monocyte-enriched peripheral blood mononuclear cells (PBMCs) and cytokine response to live and apoptotic Jurkat cells

Monocytes were isolated as described [18], [62] with some modifications. Briefly, freshly isolated PBMCs were plated in duplicates at 2×10^6^ cell/ml in RPMI containing 5% human AB serum (Sigma-Aldrich), and allowed to adhere for one hour. Non-adherent cells were removed through vigorous washing with RPMI, leaving a final concentration of approximately 1×10^6^ adherent cells, which corresponded to 80–90% CD14+ cells as determined by flow cytometry analysis (data not shown). Apoptotic Jurkat cells were generated by UVB-irradiation as previously described [2] and 5×10^6^ live or apoptotic Jurkat cells were added per well to the monocyte-enriched cells. In some experiments, monocytes and apoptotic cells were co-incubated in the absence or presence of 5 µg/ml of RNase/DNase-free, 16 u/ml of DNAse-I/RNase-free, or both nucleases (Roche Applied Science). In addition, some monocyte samples were also used for apoptotic cell clearance assays (see below). As controls, monocytes, apoptotic or live Jurkat cells were incubated alone. After overnight incubation (16–18 hrs), supernatants were collected, centrifuged to remove cellular components, and TNF-α and TGF-β were determined using a commercially available ELISA kit (R&D Systems). The optical density of each sample was determined using a microplate reader set to 450 nm (Biotrack, Pharmacia). Results were calculated from a standard curve and reported in picograms of cytokine protein per mL. Supernatants were assayed in duplicate and values were expressed as means ± standard deviations.

### Apoptotic cell clearance assay

To quantify apoptotic cell clearance by human monocytes, we established a FACS-based clearance assay. Thus, live and apoptotic Jurkat cells were carboxy-fluorescein diacetate, succinimidyl ester (CFSE)-labeled as describe by the manufacturer (Sigma-Aldrich) and the equivalent to 5×10^6^ live and apoptotic cells were co-incubated in duplicates with monocyte-enriched cells prepared as above. As controls, monocytes were incubated alone. After overnight incubation, non-adherent cells were removed by vigorous washing and the adherent monocytes were then labeled with CD14-PE (BD Biosciences, clone MφP9). The percentage of cells positive for both CD14 and CFSE was used to quantitate phagocytosis of apoptotic and live Jurkat cells by monocytes.

### Statistical Analysis

P-values were determined based on Fisher's exact test (for binary variables), Pearson Chi Square statistic (for categorical variables) and a two-sample t test (for continuous variables). In order to account for multiple comparisons between groups, the Bonferroni correction was applied.

## Supporting Information

Figure S1 Apoptotic cell clearance assay. Monocytes from healthy controls or SLE patients were co-incubated with live (**B**) or apoptotic (**C**) CFSE-labeled Jurkat cells. As controls, monocytes (**A**) or apoptotic Jurkat cells (**D**) were incubated alone. After vigorous washings, the adherent cells (**A**, **B**, and **C**) or apoptotic cells (**D**) were analyzed for CD14/CFSE positivity by FACS. We have noted that CD14 is down-regulated in monocytes incubated overnight (**A**, **B**, and **C**). A representative example from a healthy control is shown.
(TIF)

Table S1 The percentage of cells positive for both CD14 and CFSE was used to quantify phagocytosis of apoptotic Jurkat cells by monocytes. ID  =  Identification number; CFSE  =  carboxy-fluorescein diacetate, succinimidyl ester.
(DOCX)
