# Supplementary figures and images for: Abnormal Production of Pro- and Anti-Inflammatory Cytokines by Lupus Monocytes in Response to Apoptotic Cells
Source: PLoS One. 2011 Mar 14;6(3):e17495. doi: 10.1371/journal.pone.0017495 (PMC3056659; doi:10.1371/journal.pone.0017495)

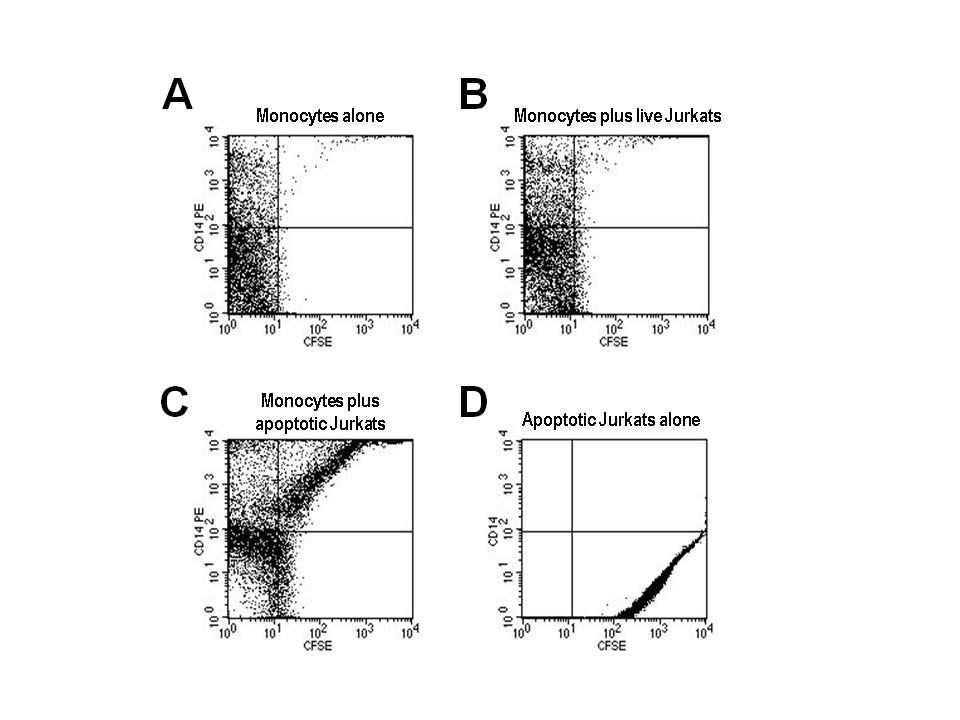

Supplement: Figure S1 — Apoptotic cell clearance assay. Monocytes from healthy controls or SLE patients were co-incubated with live (B) or apoptotic (C) CFSE-labeled Jurkat cells. As controls, monocytes (A) or apoptotic Jurkat cells (D) were incubated alone. After vigorous washings, the adherent cells (A, B, and C) or apoptotic cells (D) were analyzed for CD14/CFSE positivity by FACS. We have noted that CD14 is down-regulated in monocytes incubated overnight (A, B, and C). A representative example from a healthy control is shown. (TIF) [file pone.0017495.s001.tif]
