# Supplementary material for: Abnormal Production of Pro- and Anti-Inflammatory Cytokines by Lupus Monocytes in Response to Apoptotic Cells
Source: PLoS One. 2011 Mar 14;6(3):e17495. doi: 10.1371/journal.pone.0017495 (PMC3056659; doi:10.1371/journal.pone.0017495)
Supplement: Table S1 — The percentage of cells positive for both CD14 and CFSE was used to quantify phagocytosis of apoptotic Jurkat cells by monocytes. ID = Identification number; CFSE = carboxy-fluorescein diacetate, succinimidyl ester. (DOCX) [file pone.0017495.s002.docx]

**Supporting Information**

**Table S1.** Monocyte phagocytosis of apoptotic cells and TNF-α production

| ID | %CFSE + CD14 positive | TNF-α secretion (pg/ml) |
| --- | --- | --- |
| Control 1  Control 2  Control 3  Control 4  Control 5  Control 6  Control 7  Control 8  SLE 1  SLE 2  SLE 3  SLE 4  SLE 5  SLE 6  SLE 7  SLE 8  SLE 9  SLE 10  SLE 11  SLE 12  SLE 13  SLE 14  SLE 15  SLE 16  SLE 17  SLE 18  SLE 19  SLE 20 | 7.8  6.7  7.2  4.6  6.0  5.6  5.2  7.0  8.1  4.0  7.1  5.2  4.5  6.5  6.6  8.2  7.8  5.7  6.7  5.9  6.2  6.8  7.2  8.1  5.6  6.2  6.7  5.8 | 32  33  31  33  32  33  34  33  65  120  430  32  34  890  120  860  1000  890  330  120  65  67  32  121  66  120  330  850 |

The percentage of cells positive for both CD14 and CFSE was used to quantify phagocytosis of apoptotic Jurkat cells by monocytes.

ID = Identification number, CFSE = carboxy-fluorescein diacetate,

succinimidyl ester.
